# Autophagy During Vertebrate Development

**DOI:** 10.3390/cells1030428

**Published:** 2012-08-02

**Authors:** María R. Aburto, Juan M. Hurlé, Isabel Varela-Nieto, Marta Magariños

**Affiliations:** 1 Institute for Biomedical Research “Alberto Sols”, CSIC-UAM, C/ Arturo Duperier 4, Madrid 28029, Spain; Email: maburto@iib.uam.es (M.R.A.); 2 Unit 761, Centro de Investigación Biomédica en Red de Enfermedades Raras (CIBERER), Instituto de Salud Carlos III, Madrid 28029, Spain; 3 Departamentos de Anatomía y Biología Celular, Universidad de Cantabria, Santander 39011, Spain; Email: hurlej@unican.es (J.M.H.); 4 IdiPAZ, Madrid 28029, Spain; 5 Departamento de Biología, Universidad Autónoma de Madrid, C/ Darwin 2, Madrid 28049, Spain; Email: marta.magarinnos@uam.es (M.M.)

**Keywords:** autophagy, development, proliferation, apoptosis, cell differentiation, homeostasis, aging

## Abstract

Autophagy is an evolutionarily conserved catabolic process by which cells degrade their own components through the lysosomal machinery. In physiological conditions, the mechanism is tightly regulated and contributes to maintain a balance between synthesis and degradation in cells undergoing intense metabolic activities. Autophagy is associated with major tissue remodeling processes occurring through the embryonic, fetal and early postnatal periods of vertebrates. Here we survey current information implicating autophagy in cellular death, proliferation or differentiation in developing vertebrates. In developing systems, activation of the autophagic machinery could promote different outcomes depending on the cellular context. Autophagy is thus an extraordinary tool for the developing organs and tissues.

## 1. Introduction

**Figure 1:**
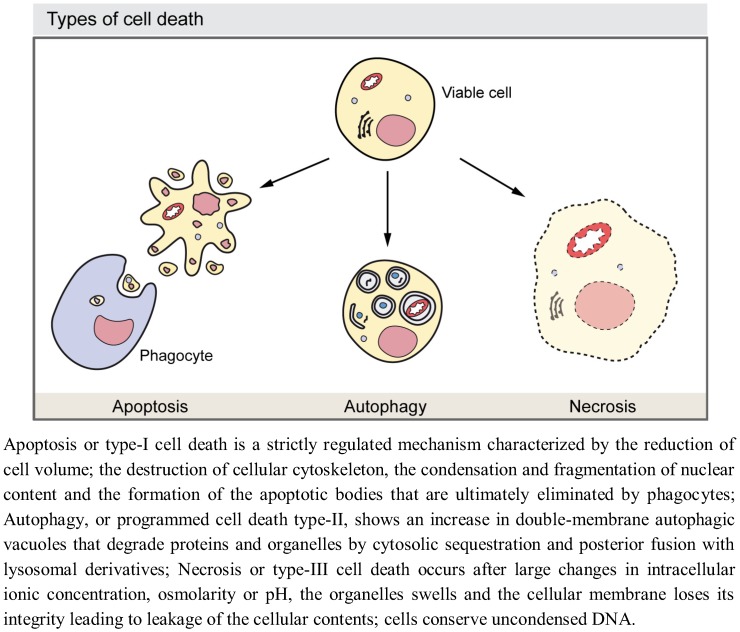
Morphological features of the major types of cell death.

Growth of developing multicellular organisms is a complex process which results from the coordination of different cellular events, including proliferation, death, and differentiation [1,2]. During all these processes individual cells often require to eliminate and/or recycle part of their own components to obtain extra energetic supply or to build new components. Autophagy is a self-degradative process first achieved by unicellular organisms to adapt to fluctuating supply of external nutrients and then also employed by multicellular organisms to account for those degradative processes [3]. In the course of development, autophagy also functions as a self-destruction mechanism responsible for the elimination of cells and/or tissues, which need to be removed. Although it has to be kept in mind that the available molecular data has changed our view of these processes (for example, it has shown ‘apoptosis’ mediated without the accepted biomarkers of apoptosis) and the existence of alternative cell death mechanisms such as entosis have been reported [4,5], historically, three major types of cell death have been described depending on the developmental context: type-I programmed cell death or apoptosis, type-II cell death or autophagy, and type-III cell death or necrosis [6,7] (Figure 1). Autophagy is observed in dying cells, especially during vast elimination of developmental tissues such as during insect metamorphosis [8]. Self-eating of cellular cytosolic constituents, though with a lower intensity, are also crucial for balancing sources of energy in response to different extracellular stimuli and for preventing the accumulation of misfolded proteins or damaged organelles. Autophagy also promotes cell survival by providing the cell with either energy or with the building blocks obtained from aged or defective macromolecules. Therefore, autophagy has been shown to have key roles in development [9,10,11], tumor suppression [12], prevention of neuron degeneration [13], anti-aging [14], and protection against intracellular pathogens and the immune inflammatory response [15,16]. Due to its functions in either eliminating molecules and organelles or providing energy or molecular components, autophagy is an important instrument to enable cellular differentiation. Accordingly, autophagy is involved in invertebrate and vertebrate development. Examples of its functions in development include the sporulation in yeast [17], the metamorphosis in flies [18], the induction of dauer arrest in worms [19], and the process of the mammalian embryo pre-implantation [20] among others [21,22].

**Figure 2:**
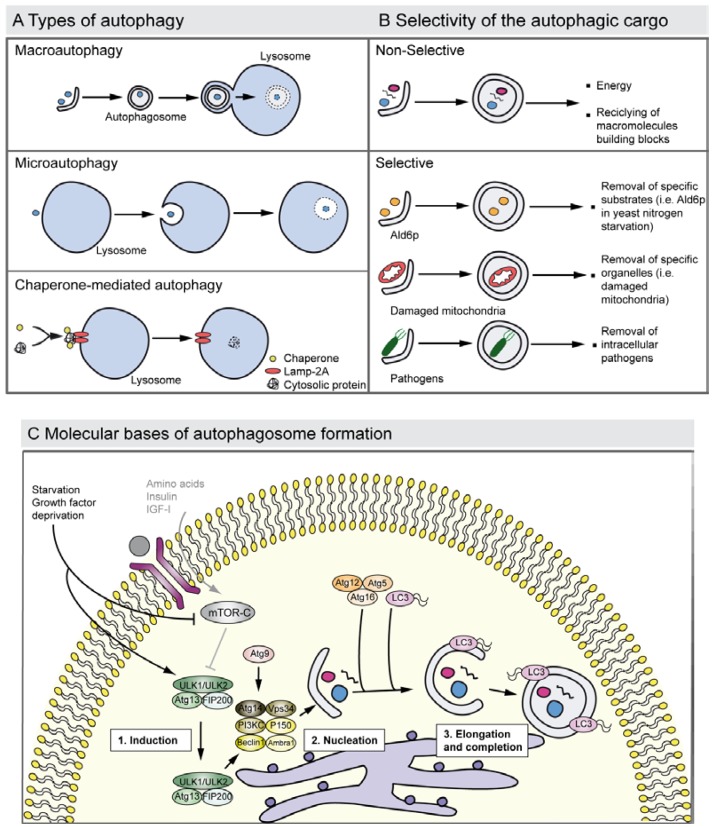
Autophagy types, cargo selectivity and the process of autophagosome formation. (**A**) The drawing shows the major types of autophagy. Macroautophagy involves the sequestration of cytosol and organelles by the autophagosome, a double membrane vacuole; the autophagosome then fuses with a lysosome and acid hydrolases degrade its contents. The process of microautophagy implicates the degradation of cytoplasmic material that is directly invaginated by the lysosome. Chaperone-mediated autophagy entails the degradation of speciﬁc cytosolic proteins that bind to chaperones and the complex joins to the lysosomal receptor Lamp-2A receptor triggering the translocation to the lumen. (**B**) Autophagy degrades selective and non-selective cargo. Autophagy may non-selectively digest fragments of cytoplasm to renovate the cellular components, or to obtain energy and free macromolecules to proceed with synthesis *de novo*. Autophagy also shows cargo selectivity degrading: (i) Specific molecules such as the elimination of the yeast Ald6p, which is negative for survival under nitrogen starvation, (ii) damaged organelles such as mitochondria with impaired membrane potential (this process is also known as mitophagy), and (iii) intracellular pathogens where it contributes to elimination of these. Selective autophagy is usually mediated by adapter proteins such as the p62 receptor and NBR1, which also function as cargo receptors in the elimination of ubiquitinated substrates. (**C**) The drawing represents a simplified overview of the factors and processes required in autophagosome formation. Growth factor deprivation or nutrient starvation regulates the translocation of the ULK1 complex to the endoplasmic reticulum and activates the phosphatidylinositol 3-kinase complex III (PI3KCIII) that promotes the nucleation of the phagophore that sequesters the cytosolic material. Atg9 is also involved in the phagophore nucleation step. Autophagosome elongation and completion is triggered by the Atg12–Atg5–Atg16 and by LC3, a homologue of the yeast Atg8 that is proteolysed into the LC3-II form and conjugates with phosphatidylethanolamine. This protein-lipid complex forms part of the double-membrane autophagosome.

Three types of autophagy have been described so far: (i) macroautophagy (herein after referred to as autophagy) that requires the formation of double membrane vacuoles, the autophagosomes, which fuse their content with lysosomes forming the autophagolysosomes; (ii) microautophagy in which the engulfment of cargo occurs by the invagination of the lysosome membrane; and (iii) chaperone-mediated autophagy that has only been reported in mammals, where there is a direct delivery of the substrates to the lysosomes [3,23,24] (Figure 2A).

Although autophagy generally degraded different constituents through an apparently arbitrary mechanism, it can also present cargo specificity and selectively to eliminate specific molecules such as the yeast cytosolic acetaldehyde dehydrogenase Ald6p that has to be removed in a nitrogen-starved medium [25] and damaged organelles such as mitochondria (mitophagy) and peroxisomes (pexophagy) [26,27] (Figure 2B). For instance, defects in the selective clearance of damaged mitochondria are implicated in the pathogenesis of Parkinson’s disease. Mitochondrial kinase PINK1 and cytosolic E3 ubiquitin ligase Parkin act in a common pathway to regulate mitochondrial function, and mutations in these genes are the most common causes of recessive Parkinson’s disease. Moreover, there is recent evidence suggesting that the PINK1/parkin pathway also plays a critical role in mitophagy. Pathogenic Parkin mutations interfere with distinct steps of mitochondrial translocation, ubiquitylation and/or final clearance through mitophagy [28,29]. Briefly, PINK1 induces the translocation of Parkin to depolarized mitochondria, which mediates the formation of two distinct poly-ubiquitin chains. The autophagic adaptor p62/SQSTM1 is then recruited to mitochondrial clusters and is essential for the clearance of mitochondria. VDAC1 (voltage-dependent anion channel 1) is also a target for Parkin-mediated poly-ubiquitylation and mitophagy [28].

The study of the function of these genes by reverse genetics has provided insight into the molecular mechanisms of autophagy and its functions in cell differentiation and organism development, both in vertebrates and invertebrates. We review here the role of autophagy in cell death, survival, proliferation and differentiation during vertebrate development. This review does not intend to be exhaustive but to focus on key examples to illustrate the importance of autophagy during embryonic development. To get further insight into aspects and animal models that are not covered here, we recommend several excellent recent reviews [11,30,31,32,33,34,35].

Autophagy begins with the formation of a phagophore that expands to form a double-membrane autophagosome that engulfs intra-cellular cargo, such as protein aggregates, organelles and ribosomes. The autophagosome fuses then with a lysosome and the acid hydrolases degrade its contents and the resulting macromolecules are recycled by the permeases. Numerous studies have revealed that there are more than 30 different autophagy-related proteins (ATG) conserved from yeast to humans [36,37] that participate in a coordinate fashion at different stages of the process [3] (Figure 2C). The serine/threonine kinase Atg1 was the first ATG identified in yeast. Vertebrate genomes encode five closely related kinases, of which UNC-51-like kinase 1 (ULK1) and ULK2 are both involved in the initiation of autophagy [38]. However the role of ULK1 in the initiation of mammalian autophagy is under discussion, as *Ulk1* knockout mice are viable and do not show any apparent autophagy related phenotype. It seems that ULK1 and ULK2 might show redundant functions, which is supported by the observed lethality of the double-knockout mice [38,39]. ULK1 is involved in mitophagy in reticulocytes [40]. ULK1 and ULK2 complexes (that include the ULK1/2, Atg13 and FIP200 proteins) are activated by AMP activated protein kinase (AMPK), which functions as an energy sensor [41]. These complexes are then translocated to the membrane of certain regions of different intracellular organelles that will form an expanding membrane structure: the phagophore. The source of the membrane that generates the phagophore is under intense debate. Potential membrane origins include the endoplasmic reticulum, Golgi complex, mitochondria, endosomes and the plasma membrane [42]. The autophagic stimulus also contributes to the membrane source as has been proposed with mitochondria and its central role in starvation-induced autophagy [43]. In mammalian cells there are two types of phosphatidylinositol 3-kinases (PI3K): Class I and Class III. The Class III PI3K is known to participate in various membrane trafficking events and it forms a complex, the PI3KCIII, in which the core proteins are Beclin-1, Atg14, p150 and Vps34. Amino acid deprivation leads to autophagy activation through mTOR inhibition. In that sense, a major pathway by which amino acids control mTOR is mediated through the Class III PI3K, through the regulating actions of the ULK complexes. On the other hand, Class I PI3K acts through an insulin signaling cascade to activate mTOR and PKB; hence it has an inhibitory effect on autophagy [44]. Autophagy is positively and negatively regulated through Beclin-1 interactions by UVRAG and Rubicon, respectively [45,46,47]. Ambra-1, the product of a gene only found in vertebrates, also positively regulates autophagy by promoting Beclin-1 interaction with Vps34 [48]. Atg9 is the only known transmembrane protein essential in the autophagy pathway. Atg9 is involved in the autophagosome biogenesis, acting as a membrane deliverer that cycles between membrane organelles but does not stably integrate on the autophagosome [49,50]. Autophagosome elongation requires evolutionary conserved ubiquitin-like conjugation systems and is carried out by lipidic modifications by the action of phosphatidylethanolamine (PE) of microtubule-associate protein 1 light chain 3 (MAP1LC3/LC3), the mammalian homolog of Atg8 in yeast [51]. This process is orchestrated, among others, by Atg7 (an E1-like ubiquitin conjugating enzyme), Atg3 (an E2-like ubiquitin conjugatin enzyme [52]), and Atg4C, to which LC3 is bound at first. Atg7 also acts in an ubiquitin-like conjugation system involving the E2-like ubiquitin enzyme, Atg10 and Atg12/Atg5, which, at the end of the process, are transferred to Atg16L. The complex Atg12/Atg5/Atg16L mediates LC3-PE by binding to the autophagosome membranes [53,54] and promotes the elongation and isolation of the autophagosome [3,30] (Figure 2C). Atg4/autophagin cleaves LC3 into its cytosolic version, also known as LC3-I. LC3-I generation is started by Atg7, transferred to Atg3, and finally modified with a lipidic attachment to bind with the autophagosome membrane, constituting the membrane-bound form LC3-II [55,56,57]. This conversion is considered as a hallmark to detect active autophagy (Figure 2C). Both LC3 and Atg4 proteins have been genetically abrogated in mice [58,59], and interestingly, none of them showed developmental abnormalities, which probably implies redundancy in the Atg4 family and the existence of at least two murine forms of LC3 (LC3α and LC3β) [58].

## 2. Autophagy as a Cell Death Mechanism

Basal autophagy is a survival process that contributes to cell homeostasis. Autophagy acts as a fast-response pathway against nutrient deprivation or oxidative stress, favoring cell homeostasis. In addition, autophagy can also constitute a cell death mechanism, namely type-II cell death or autophagic cell death. It is characterized by an increased number of large autophagic vacuoles that digest cytoplasmic material, and also by its independence of phagocytosis [7]. However, the role of autophagy as a cell death mechanism is a controversial issue, specifically in the mammalian system [60]. The presence of autophagosomes in dying cells has been taken as a feature of cell death, but it may represent an epiphenomenon that coexists with cell death [61,62]. Autophagosomes can be an initial attempt of the cells to survive, thus, a damaged cell might trigger autophagy as a protective mechanism, but finally die. Therefore, vacuolated dead cells may die with autophagy, but not by autophagy [60]. However, autophagy might ultimately cause cell death by massive cytosolic self-digestion or by selective elimination of protective proteins such as the catalase [63]. Even considering the presence of autophagosomes in dying cells as a controversial subject, autophagy as a cell death mechanism has been demonstrated in lower eukaryotes, where there are many examples. For example in *Dictyostelium discoideum*, where the absence of the apoptotic machinery facilitates the understanding of the molecular autophagic pathway [64], and in *Drosophila melanogaster. Drosophila* larvae experience large cellular remodeling during metamorphosis to achieve tissue maturation: Several structures such as the fat body and the salivary glands have to be degraded to generate the adult organism. Loss of function mutations in *Atg* genes shows the permanence of the glands for at least 24 h longer [65], which provides evidence for the role of autophagy in developmental cell death. These events are regulated by an increase in the levels of the hormone ecdysone, which triggers the autophagic program in a variety of tissues, which starts with the rise of some ATG mRNA levels [66]. Mutant flies in *Atg1*, *Atg2* or *Atg18* show that the elimination of midgut cells is also dependent on autophagy [67]. However, the possibility that autophagy is acting in a non-cell autonomous fashion has to still be excluded, and thus the cells that show autophagy markers are not the cells that will eventually die.

Although autophagic cell death is observed in mammalian cells in culture [68] and accompanies other degradation processes in areas of massive cell death during development [21], cell death *by* autophagy is infrequent in vertebrates [60]. The lack of cell death defects in distinct autophagy-deficient mutant mice raises the question of whether autophagy *per se* or only part of the autophagy pathway is involved in type-II cell death during development [59,69,70]. Due to its high adjustability, apoptosis might be the manager of removing individual cells while autophagy might be useful to remove high quantities of tissues as required in larval metamorphosis [5].

Additionally, autophagy and apoptosis may contribute together to cell elimination and indeed they are strictly connected. Thus, a dying cell may show features of both types I and II cell death, as for example, the HIV-infected CD4+ T lymphocytes that undergo apoptotic cell death induced by autophagy [71]. Autophagy can also assume the killer role when apoptosis is unavailable. For example, autophagy mediates cell death in apoptosis-deficient BAX^−/−^ BAK^–/–^ cells in response to genotoxic or endoplasmic reticulum stress stimuli [72,73,74]. In some cases, autophagy might ultimately cause cell death by massive cytosolic self-digestion in response to cellular stress during apoptosis-deficient conditions. Related to this, it is interesting to point out that autophagy and apoptosis are strongly connected through the BH3-only proteins, Bcl-2 and Beclin-1. Bcl-2 is an antiapoptotic protein, a key player of the intrinsic apoptotic pathway that regulates mitochondrial permeabilization. Beclin-1 has a BH3 domain [75] that interacts with Bcl-2, and this interaction prevents the initiation of autophagy [72,76], which reflects the convergent regulation of apoptosis and autophagic cell death [77]. Relative amounts of Beclin-1 and Bcl-2 seem to regulate the transition from cell homeostasis to cell death. Accordingly, the absence of autophagy genes increases cell death during nutrient deprivation and other forms of cellular stress [10]. Such studies raise the possibility that autophagic cell death might be induced in a similar manner to that of apoptosis. Remarkably, there is increasingly more data showing the crosstalk of apoptosis and autophagy and the dual-role of their components. Indeed, many core proteins of one of the processes have been reported to regulate the other. For example, caspases inhibit autophagy by the cleavage of Beclin-1 and Vsp 34 [78,79,80]. The recent role of Atg12 in this apoptosis-autophagy crosstalk is also very interesting. Atg12 acts as a positive apoptosis regulator by interacting with members of the Bcl-2 family by a predicted BH3-like motif [81].

Autophagy has also been reported to be essential for apoptosis by providing energy for phosphatidylserine (PS) exposure in the outer leaflet of the apoptotic cell plasma membrane. Apoptosis in mammals is initiated upon different upstream signals that lead to the activation of members of the Bcl-2 family that in turn inhibit anti-apoptotic members, such as Bcl-2, BCL-XL and MCL1 by direct interaction in the outer mitochondrial membrane. This causes the release of the BAX and BAK protein inhibition, which in turn leads to mitochondrial damage and cytochrome c release. Cytochrome c promotes the formation of the apoptosome, composed of APAF1 and caspase-9, which cleaves and activates downstream caspases, including caspase-3, caspase-6 and caspase-7 that carry out the execution phase of apoptosis [82]. Apoptotic cells need to be actively eliminated from the surrounding living cells, and it is known that PS exposure on the cell surface is an ‘eat me’ signal, which triggers engulfment by phagocytes that efficiently remove PS expressing cells. Thus, in this context, autophagy facilitates the clearance of the apoptotic bodies [83,84,85]. Thus, the interaction Beclin-1-Bcl-2 represents a molecular switch between apoptosis and autophagy.

**Figure 3:**
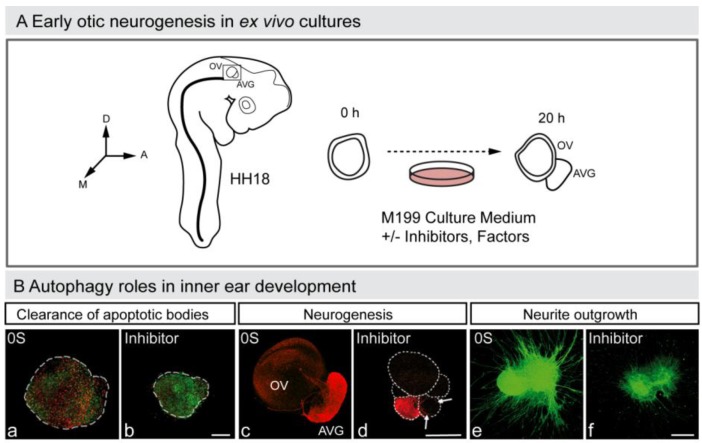
Autophagy in early otic neurogenesis. (**A**) Schematic representation of the otic vesicle *ex vivo* culture. The otic vesicle can be explanted from the embryo at HH18. The acoustic-vestibular ganglion also develops *ex vivo*, and thus this constitutes an excellent model to study otic neurogenesis. The figure shows a schematic drawing of a HH18 chicken embryo showing the location of the otic vesicle, of an otic vesicle immediately after dissection (0 h) and after 20 h in culture (20 h). Factors and drugs can be added to the serum-free culture medium to study their effects on the otic vesicle’s *ex vivo* development and AVG formation. Abbreviations: AVG, acoustic-vestibular ganglion; OV, otic vesicle. (**B**) Autophagy and inner ear development. Otic vesicles were incubated with or without autophagy inhibitor and then labeled to detect apoptotic cells with annexin-V (An-V, red) and TUNEL (green) (a,b), or to study neurogenesis (red; c,d). The AVG can also be cultured and labeled to study axon outgrowth (green; e,f). Orientation: A, anterior; D, dorsal; M, medial. (Adapted from [88]).

In summary, autophagy in vertebrate development, besides being responsible for cell elimination in the few examples already mentioned, above all accompanies programmed cell death. Moreover, autophagy provides energy for the clearance of apoptotic bodies during mouse embryonic cavitation [85], a role also confirmed in cultures of developing avian retina [22]. The interplay between autophagy and apoptosis is an emerging aspect of developmental biology [86], which has a particular relevance for otic neurogenesis during early inner ear development (Aburto, unpublished observation; Figure 3) [87,88].

## 3. Autophagy in Cell Cycle Regulation

Numerous observations suggest that autophagy is strongly associated with cell cycle regulation. The deficiency in autophagy genes causes the miss-regulation of cell proliferation, for example, *Atg4C* [59], *Atg5* [89], *Bif-1* (Bax-interacting factor 1) a positive inductor of PI3KCIII and of autophagy in mammalian cells) [90] or *Beclin-1* [91,92], that triggers mutations and tumors in the mouse. Miss-regulation of cell cycle due to defects in autophagy has also been observed during development in the *Ambra1* mutant mice and in early otic neurogenesis through pharmacologic inhibition (Aburto, unpublished observation; Figure 3). The *Ambra1* deficient mouse embryo exhibits increased proliferation in the neuroepithelium, excessive apoptosis and defects in neural tube closure [48]. These data indicate that Ambra1 is required in cell cycle regulation during nervous system development. In physiological conditions, the complex Ambra1-Beclin-1 is anchored to the microtubule machinery through dynein, whereas under nutrient deprivation when autophagy is induced, the complex is released [93]. Organotypic cultures of otocysts exposed to chemical inhibitors of autophagy show an increase in the fraction of cells that progress through the G_1_/S-phase checkpoint but that are unable to complete cell division (Aburto, unpublished observation). Treated otocysts also showed a remarkable accumulation of apoptotic cells that might be due to a failure in cell cycle regulation during inner ear development. Autophagy has an important role in recycling proteins and, thus, essential negative-cell cycle regulators may not be available under autophagy impairment. Additionally, differentiation processes also require cytosolic rearrangements. Without autophagy, developing progenitors may not progress through the differentiation program and thus they could initiate a new unnecessary proliferation round. Moreover, defects in autophagy could deregulate the dynamics of microtubules, which are essential to complete the cell cycle [10]. Considering the high incidence of tumors in mutants of autophagic genes and the important role of cell proliferation in development, there is an emerging interest in understanding the involvement of autophagy in cell cycle regulation and cancer [94,95].

## 4. Autophagy in Cell Differentiation and Development

Cell differentiation and functional specification are sequentially acquired during development once the proper number of cells has been generated. Autophagy during this developmental stage may facilitate rapid changes in cytosolic composition, accelerating protein and organelle turnover and the recycling of specific factors exposed receptors and cytoskeletal dynamics necessary to promote the different cell fates. By promoting autophagy, cytosolic composition can be rapidly modulated in the differentiating cells, facilitating alternative pathways for protein and organelle rearrangement. Moreover, it constitutes an extra source of energy supplied by the self-degradation of the cytoplasmic material. Autophagy is crucial for vertebrate development at specific time points of embryogenesis. Immediately after birth, trans-placental nutrient supply is suddenly interrupted, and neonates face severe starvation until supply can be restored through milk nutrients. In mice, the level of autophagy remains low during embryogenesis; however, it is immediately up-regulated in various tissues after birth and is maintained at high levels for 3–12 h before returning to basal levels within 1–2 days. Regarding that, mice deficient in Atg5 appear almost normal at birth, but die within one day after delivery. These mice exhibit reduced amino acid concentrations in plasma and tissues and display signs of energy depletion, which highlights the importance of autophagy in the maintenance of energy homeostasis during the neonatal starvation period [96].

There are data supporting that autophagy is required for elimination of paternal mitochondria in *Caenorhabditis elegans* after oocyte fertilization, where immediately after fertilization, sperm-derived components trigger localized induction of autophagy around sperm mitochondria, which leads to its degradation and consequently to the exclusive maternal inheritance of mitochondrial DNA [97,98].

**Figure 4:**
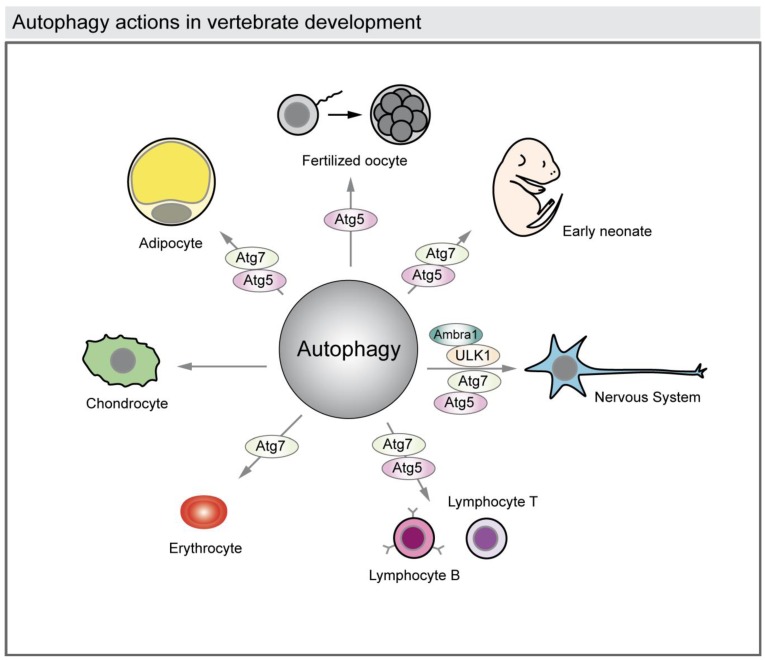
Autophagy actions in vertebrate development. The illustration represents the reported functions of autophagy in vertebrate development. Autophagy is required to rearrange the cytosolic composition of fertilized oocytes and to provide energy and building blocks of macromolecules to the newborns during the first days. Autophagy also acts in cell differentiation in the developing nervous system, heart, osseous and adipose tissues, and also during hematopoiesis (lymphocytes and erythrocytes). The reported autophagy-related proteins (ATG) genes involved in those processes are shown.

Autophagy has also been associated with cell differentiation during development of several tissues such as the nervous system, the heart, the hematopoietic system, osseous tissue, and adipose tissue (Figure 4) [11,31].

### 4.1. Autophagy Facilitates Remodeling at Specific Times of Development

Oocytes have maternal mRNA and proteins that direct their progression until fertilization takes place and they become a zygote. After fertilization, the oocyte changes its cytoplasm composition by using the autophagy machinery to remodel its transcript and protein content and proceed with the zygotic program that will form the embryo [99,100]. Thus, although *Atg5^−/−^* null mice manage to survive up to early post-natal ages, the oocyte-specific elimination of maternal inherited *Atg5* causes embryonic death at the four- to eight-cell stage [20].

Mammalian embryos obtain energy through the placenta. After birth, there is a period during which embryos face starvation before the suckling behavior is established. Accordingly, autophagy is induced during 3–12 h after birth in several tissues [96]. Several mutant mice in ATG proteins die during this fragile perinatal period. In addition to the *Atg5^−/−^* mouse, mutants in the genes *Atg3*, *Atg7*, *Atg9* and *Atg16L1* die soon after birth although they show a healthy appearance [69,101,102,103]. In this context, autophagy has been proposed to be a source of energy, based on the fact that these mutants show reduced amino-acid blood levels [69,96,103] and adipose tissue [104]. Moreover, defective clearance of the apoptotic bodies in *Atg5^−/−^* mice may also contribute to early lethality [85]. Finally, impaired autophagy in the nervous system of *Atg5* and *Atg7* deficient neonates affects suckling behavior, potentiating tissue starvation [96].

### 4.2. Autophagy in the Developing Nervous System

Autophagy contributes to nervous system development at different levels. Impaired autophagy causes uneven numbers of neural progenitors and, later in development, it may impair the competence of neural populations to transform the cytosolic milieu and differentiate properly. This may include axonal outgrowth or the expression of the precise neurotransmitter. Defects reported in *Ambra1* null mice might reflect the first possibility. Ambra1 is a vertebrate-only protein highly expressed in the nervous system that binds to Beclin-1. *Ambra1*^−/−^ mice show hyper-proliferation of neuroepithelial cells, increased apoptosis and display brain patterning defects [48]. An example of the impairment caused in terminal neuronal differentiation by defects in autophagy is shown by the phenotype of *Ulk1*^−/−^ mice. The deficiency in ULK1, an ATG protein involved in the initiation of the autophagosome, causes abnormal axonal formation in the cerebellar granule neurons [105]. UNC-51, the ortholog of ULK1 in *Caenorhabditis elegans* and in *Drosophila melanogaster,* is also involved in neurite outgrowth [106,107]. Inner ear developmental defects due to mutations in ATG genes have also been reported in mice [108], and *Ifg1*^−/−^ mice show miss-regulation of several autophagic genes in the cochlea [109]. Accordingly, during early chicken inner ear development autophagy has a key role in otic neurogenesis and, interestingly, it is also involved in axonal outgrowth (Figure 3; Aburto, unpublished observations). Besides cellular phenotypes, defects in autophagy during nervous system development also have behavioral consequences, for example, *Atg5* and *Atg7* mutant mice show suckling defects [96] and have decreased motor function [110].

### 4.3. Autophagy During Hematopoiesis: Lymphocytes and Erythrocytes

To study the functions of autophagy in hematopoietic cells, Pua *et al*. generated mouse chimeras by transferring fetal liver cells from *Atg5^−/− ^*mice after irradiation [111]. This study showed that defects in autophagy led to a reduction of thymocytes and B-lymphocytes. These results suggested that Atg5 regulates lymphocyte development and function. The haematopoietic cell-specific *Atg7^−/−^* mice showed that Atg7 was also implicated in T-lymphocyte development [112]. These T-cells present a higher mitochondrial content which facilitates the initiation of the apoptotic program. During T-cell development, the number of mitochondria is reduced; however, if autophagy is impaired, mitophagy is suppressed and concomitantly T-cell viability decreases [113].

The study of the haematopoietic cell-specific *Atg7*-deficient mice also showed that erythrocytes depended on autophagy for their correct development [112]. In order to bring oxygen to all the cells of the organism, erythrocytes need to fit in capillaries with very small diameters. To this end, erythroblasts first lose their nuclei and become reticulocytes, which then lose the rest of their organelles to give rise to the erythrocytes. Mice with *Atg7-*deficient erythroblasts accumulate mitochondrial masses and do not proceed with the maturation program. Thus, autophagy promotes mitochondria removal during erythrocyte differentiation. 

### 4.4. Autophagy in Developing Osseous Tissue

Osseous tissue, or bone tissue, forms the rigid part of the bone organs. Osteoblast formation from chondrocytes is a process that remains largely unknown. Chondrocytes produce and maintain the cartilaginous matrix, an environment with reduced levels of nutrients and oxygen. Autophagy has been proposed to serve a dual function, first by providing the energy that chondrocytes require to live in such a deprived microenvironment, and second by eliminating the terminal chondrocytes during bone formation [114].

### 4.5. Autophagy in Developing Adipose Tissue

Autophagy has a very interesting role in the regulation of adipocyte differentiation. Adipocytes are cells whose cytoplasm is mainly occupied by a large lipid droplet. In addition to the classic pathway of lipid metabolism by cytosolic lipases, lipid droplets have been identified as a substrate for macroautophagy. Lipid droplets are sequestered in autophagosomes for the breakdown of the lipidic components, which allows the cell to further respond to energy demands [115].

The nucleus and the rest of organelles in adipocytes are located in the small volume left. Adipocytes differentiate from mesenchymal precursors and undergo an intense remodeling process to become a fat depot. The differentiation of white adopocytes is accompanied by an initial increase in mitochondria biogenesis followed by a vast reduction in the number of mitochondria in the cell, which are predominantly substituted by the lipid droplet. It has been documented that there is a massive autophagy activation and engulfment of mitochondria by autophagosomes during adipogenesis [116]. The differentiation events can be mimicked by the addition of specific adipogenic factors to mouse embryonic fibroblasts (MEF). In this context, it has been reported that MEFs deficient in *Atg5* show reduced efficiency in adipocyte differentiation. They also observed that in vivo *Atg5^−/−^* embryos had reduced subcutaneous adipose mass [104]. The genetic inhibition of *Atg7* in pre-adipocytes inhibited lipid accumulation in a single lipid droplet and promoted an accumulation of mitochondria [117]. Moreover, the adipocytic cell-type specific *Atg7* knockout has less adipose mass and its white adipose tissue shows features of brown adipose tissue [117,118]. The relative equilibrium of white to brown fat is related to body weight [119]. It is also worth noting that suppression of *Atg7* in the hypothalamus induces obesity [120]. These studies place autophagy as a key process for adipocyte differentiation and, more importantly, with a central role in the development of obesity.

## 5. Autophagy in Tissue Homeostasis and Aging

Basal autophagy has an important role in adult post-mitotic cells as a “quality control mechanism” that protects cell survival by refreshing the cytosolic content. Abnormal protein turnover promotes the generation of protein aggregates that need to be eliminated. Macroautophagy is responsible for removing protein aggregates, while chaperon-mediated autophagy is involved in lysosomal degradation of soluble misfolded or transformed proteins [121,122]. By autophagy, damaged organelles such as mitochondria and peroxisomes, are also removed [123,124]. Thus, autophagy acts as a physiological mechanism against neurodegeneration, cancer and several infections [34,125,126]. Accordingly, loss of *Atg5* or *Atg7* in neurons and cardiomyocytes shows increased poly-ubiquitinated aggregates [69,110]. Moreover, autophagy is a protective mechanism for the cell against metabolic stress, particularly against hypoxia, nutrient starvation and growth factor reduction. Lysosomal degradation serves as a recycling mechanism that provides the cell with free amino acids or fatty acids that can be used for *de novo* synthesis or to obtain ATP [127,128].

In summary, autophagy plays an important cytoprotective role in basal conditions by the elimination of aberrant proteins and organelles and in consequence, there is a remarkable connection between autophagy malfunction, ageing and disease [14]. Not surprisingly, mechanisms that promote life-span extension also induce autophagy, including caloric restriction, NAD-dependent deacetylase sirtuin-1 activation, p53 suppression, inhibition of insulin/IGF-1 actions, rapamycin-mediated inhibition of mTOR, and treatment with spermidine or resveratrol [14,129,130]. Therefore, autophagy can be envisaged as a novel anti-aging mechanism, but further work is needed to clarify if it is a cause or a consequence.

## 6. Conclusions

Current evidence indicates that autophagy contributes to programmed cell death, proliferation, survival and differentiation to modulate cell number and fate during vertebrate development. Autophagy is fundamental after oocyte fertilization, at early neonatal stages and for the differentiation of a variety of tissues. Autophagy is also essential for tissue homeostasis and its deregulation has been associated with human ageing and diseases. Future work will provide insight into novel functions and mechanisms of action, which would help the design of novel strategies against autophagy-related diseases.
